# *Nauclea latifolia* Sm. Leaf Extracts Extenuates Free Radicals, Inflammation, and Diabetes-Linked Enzymes

**DOI:** 10.1155/2020/5612486

**Published:** 2020-03-09

**Authors:** Franklyn Nonso Iheagwam, Emmanuel Nsedu Israel, Kazeem Oyindamola Kayode, Opeyemi Christianah DeCampos, Olubanke Olujoke Ogunlana, Shalom Nwodo Chinedu

**Affiliations:** ^1^Department of Biochemistry, Covenant University, Canaanland, P.M.B, 1023 Ota, Ogun State, Nigeria; ^2^Covenant University Public Health and Wellbeing Research Cluster (CUPHWERC), Covenant University, Canaanland, P.M.B, 1023 Ota, Ogun State, Nigeria

## Abstract

This study was carried out to assess the *in vitro* antioxidant, anti-inflammatory and antidiabetic effects of *Nauclea latifolia* (Sm.) leaf extracts. Ethanolic (NLE) and aqueous (NLA) extract of *N. latifolia* leaves were prepared and assessed for their anti-inflammatory activity, antioxidant potential, *α*-amylase and *α*-glucosidase inhibitory activities, and the mechanism of enzyme inhibition *in vitro* using standard established methods. From the results, phytochemicals such as flavonoids, phenolics, glycosides, and tannins were detected in both extracts of *N. latifolia* with NLE having a significantly (*p* < 0.05) higher phytochemical content. NLE displayed significantly (*p* < 0.05) better total antioxidant capacity, reducing power, 2,2-diphenyl-2-picrylhydrazyl, and hydrogen peroxide radical scavenging activities. For anti-inflammatory activities, 70.54 ± 2.45% albumin denaturation inhibition was observed for NLE while 68.05 ± 1.03% was recorded for NLA. Likewise, 16.07 ± 1.60 and 14.08 ± 1.76% were obtained against hypotonic solution and heat-induced erythrocyte haemolysis, respectively, for NLE while 20.59 ± 4.60 and 24.07 ± 1.60% were respective NLA values. NLE (IC_50_: 4.20 ± 0.18 and 1.19 ± 0.11 mg/mL) and NLA (IC_50_: 11.21 ± 0.35 and 2.64 ± 0.48 mg/mL) *α*-glucosidase and *α*-amylase inhibitory activities were dose-dependent with uncompetitive and competitive inhibition elicited, respectively, by the extracts. A significant positive association (*p* < 0.01 and 0.05) was identified between antioxidant activity and carbohydrate-metabolising enzyme inhibitory activity. The obtained result suggests *N. latifolia* leaf could serve as an alternative candidate for managing diabetes mellitus due to its antioxidant and anti-inflammatory association with diabetes-linked enzymes.

## 1. Introduction

Diabetes mellitus (DM) is a noncommunicable, chronic ailment that is not only affecting a high proportion of the world's populace but also affecting more of the developing countries of the world compared to the developed nations [1]. A worldwide survey by International Diabetes Foundation (IDF) showed a diagnosis of 415 million people with diabetes, with a projected increase to over 600 million people by 2040 [2]. Epidemiological statistics show that Nigeria is responsible for a fifth of all reported cases of diabetes in the sub-Saharan Africa, with a steep increase in the prevalence of this disease from the rural area to members of the high socioeconomic population [3]. *Nauclea latifolia* is an evergreen tree with multiple stems and adapts very well in both the tropical rainforest zone and the savanna woodlands situated in the west and central Africa [4]. This tree is known to have various medical uses, particularly by folk medicine men [5]. It can also serve as a chewing stick to treat stomachache and tuberculosis at its initial stage [6]. The following are some medical conditions that have been treated using *N. latifolia*: wounds, cough, gonorrhoea, malaria, and hypertension [7]. Reports show that *N. latifolia* formulation and decoction preparations are used in ethnomedicine to treat hyperglycaemia and diabetes by different ethnic groups in Nigeria [8]. Nevertheless, there is little information on the inhibitory activity of *N. latifolia* leaf extract on *α*-glucosidase and *α*-amylase, and the mechanisms are yet to be experimentally documented. The association between the inhibition of these enzymes and its reported antioxidant activities has also not been examined. These observations prompted this study to assess the antidiabetic mechanisms of *N. latifolia* leaf extracts on enzymes linked to diabetes mellitus. The antioxidant and anti-inflammatory activities were assessed as well as the correlation between these activities with the antidiabetic property.

## 2. Material and Methods

### 2.1. Chemicals and Reagents

Rat intestinal *α*-glucosidase and porcine pancreatic *α*-amylase were procured from Solarbio, China. Hydrogen peroxide, butylated hydroxytoluene (BHT), acarbose, and para-nitrophenyl-glucopyranoside (*ρ*NPG) were obtained from Sigma-Aldrich, USA. Amylose, other chemicals, and reagents used were of analytical grade.

### 2.2. Collection and Identification of Plant Samples

Healthy *Nauclea latifolia* leaves were acquired in November 2016, from Ibadan in Oyo State, Nigeria and identified by Dr. J. O. Popoola of Covenant University, Nigeria. The plant sample was deposited in the herbarium where voucher (NL/CUBio/H810) and identification (FHI 112779) numbers were assigned.

### 2.3. Collection of Blood Samples

Human erythrocytes (3-5 mL) were collected from healthy volunteers (18-20 years) without a history of anti-inflammatory drugs for at least two (2) weeks. Participants were briefed on the study, and their consent was required for participation. Covenant University Health Research and Ethics Committee granted ethical approval (CHREC/031/2018) for the study as guidelines of the Declaration of Helsinki were strictly adhered.

### 2.4. Extract Preparations

*N. latifolia* leaves were dried at room temperature for 14 days and pulverised to a uniform size. The grounded leave sample (100 g) was steeped in 1 L of ethanol (80%) and distilled water for three (3) days and filtered. The obtained filtrate was concentrated using a rotary evaporator (Stuart, RE 300/MS, Staffordshire, UK) set at 50°C and 60°C to yield a greenish and brownish crude paste for ethanol (NLE) and aqueous (NLA) extracts, respectively [9].

### 2.5. Phytochemical Analysis

#### 2.5.1. Qualitative Phytochemical Analysis

The standard tests for flavonoids, alkaloids, anthocyanins, tannins, cardiac glycosides, terpenoids, triterpenoids, saponins, betacyanins, quinones, glycosides, phenols, and coumarins were carried out using standard methods described by Varadharajan, Janarthanan, and Krishnamurty [10].

*(1) Tests for Tannins*. Two mL of ferric chloride (5%) was added to 1 mL of sample in a tube. The presence of tannins in the sample was indicated by the formation of greenish-black colour.

*(2) Test for Saponins*. Equal portions (2 mL) of both sample and distilled water were added in a tube and shaken lengthwise for 15 min. The presence of saponins in the sample was indicated by the formation of a 1 cm layer of foam.

*(3) Test for Flavonoids*. To 10 mL of sample, 5 mL of dilute ammonia solution was initially added before 5 mL of concentrated sulphuric acid was after that added. The presence of flavonoids in the sample was indicated by the formation of yellow colouration.

*(4) Test for Alkaloids*. Two mL of concentrated hydrochloric acid was added to 2 mL of sample. Then, a few drops of Mayer's reagent were added to the mixture. The presence of alkaloids in the sample was indicated by the formation of a green colour.

*(5) Test for Betacyanins*. One mL of 2 N sodium hydroxide was added to 2 mL of sample and incubated for 5 min at 100°C. The presence of betacyanins in the sample was indicated by the formation of a yellow colour.

*(6) Test for Anthocyanins*. The presence of anthocyanins was demonstrated by adding 2 mL of 2 N hydrochloric acid to 2 mL of sample. The appearance of a pink-red colour that turns purplish-blue after addition of ammonia indicates the presence anthocyanins.

*(7) Test for Quinones*. One mL of concentrated sulphuric acid was added to 1 mL of sample. The presence of quinones in the sample was indicated by the formation of a red colour.

*(8) Test for Glycosides*. To 2 mL of sample, 3 mL of chloroform was added. Thereafter, 10% ammonia solution was added to the mixture dropwise. The presence of glycosides in the sample was indicated by the formation of a pink colour.

*(9) Test for Cardiac Glycosides*. Glacial acetic acid (2 mL) and few drops of ferric chloride (5%) were added to 500 *μ*L of the sample. One mL of concentrated sulphuric acid was added to underlay the mixture. The presence of cardiac glycosides in the sample was indicated by the formation of brown ring formation at the interface.

*(10) Test for Terpenoids*. Two mL of chloroform was added to 500 *μ*L of the sample before concentrated sulphuric acid was carefully added to the mixture. The presence of terpenoids in the sample was indicated by the formation of reddish-brown colour.

*(11) Test for Triterpenoids*. One mL of Liebermann-Burchard reagent (made up of acetic anhydride and concentrated sulphuric acid in ratio 1 : 1 *v*/*v*) was added to 1.5 mL of sample. The presence of triterpenoids in the sample was indicated by the formation of a blue-green colour.

*(12) Test for Phenols*. Two mL of distilled water was added to 1 mL of sample. Thereafter, 10% ferric chloride was added to the mixture dropwise. The presence of phenols in the sample was indicated by the formation of a green colour.

*(13) Test for Coumarins*. One mL of 10% sodium hydroxide was added to 1 mL of sample. The presence of coumarins in the sample was indicated by the formation of a yellow colour.

#### 2.5.2. Quantitative Phytochemical Analysis

*(1) Total Phenolic Content (TPC) Assessment*. TPC was determined according to the Folin-Ciocalteu method as described by Sharma, Goyal, and Sharma [11]. Gallic acid (GA) standard curve was estimated via preparation of GA solution in 80% methanol. Briefly, to 100 *μ*L of the sample, Folin-Ciocalteu reagent was added (ratio of 1 : 10), mixed, and incubated at 25-37°C for 1 min. Subsequently, 1.5 mL of 20% sodium carbonate was added to the mixture, shaken, and incubated for 90 min in the dark at room temperature. The absorbance was afterwards taken at 725 nm while the total phenolic content was expressed as mg of gallic acid equivalents per g of extract (GAE/g).

*(2) Total Flavonoid Content (TFC) Assessment*. TFC was determined by aluminium chloride assay as described by Sharma et al. [11]. In a tube, 500 *μ*L of the sample was first added to 2 mL of distilled water. Afterwards, 150 *μ*L of 5% sodium nitrite was added and incubated for 5 min. Thereafter, 150 *μ*L of 10% aluminium trichloride was added to the mixture prior to incubation for 6 min. Two mL of 4% sodium hydroxide was later added, and the mixture was incubated for 15 min to allow for pink colour development of the mixture. The absorbance was afterwards taken at 510 nm while the total flavonoid content was expressed as mg of rutin equivalents per g of extract (RE/g).

*(3) Total Tannin Content (TTC) Assessment*. TTC was determined by slightly modifying the Folin-Ciocalteu method as described by Tufikul et al. [12]. A volume of 100 *μ*L of standard (tannic acid) (6.25, 12.5, 25, 50, 100, and 200 *μ*g/mL) and sample (200 *μ*g/mL) solution were taken in different sample tubes, to which 500 *μ*L of Folin phenol reagent, 1 mL of sodium carbonate (35%) solution, and 7.9 mL of distilled water were added. The mixture was shaken, incubated at room temperature for 30 min, and absorbance was measured at 725 nm against a blank. TTC was expressed as mg tannic acid equivalent per gram of extract (TAE/g).

*(4) Beta-Carotene (β-Carotene) and Lycopene Assessment*. *β*-Carotene and lycopene content were determined according to the spectrophotometric method described by Sharma et al. [11]. Ten mL of acetone-hexane mixture (4 : 6) was added to 1 mL of sample, vigorously shaken for 1 min, and filtered to yield a filtrate. The absorbance of the filtrate was measured at 453, 505, 645, and 663 nm. The content of *β*-carotene and lycopene was calculated using the formula in equations (1) and (2), respectively:

(1) $$\begin{array}{c} \beta -\text{carotene }\left(\text{mg}/100\text{ mL}\right)=0.216{\text{A}}_{663}–0.304{\text{A}}_{505}+0.452{\text{A}}_{453}, \end{array}$$

(2) $$\begin{array}{c} \text{Lycopene }\left(\text{mg}/100\text{ mL}\right)=-0.0458{\text{A}}_{663}+0.372{\text{A}}_{505}+0.0806{\text{A}}_{453}. \end{array}$$

The values were expressed as *μ*g carotene equivalent per g of extract (*μ*g CAE/g).

*(5) Total Alkaloid Assessment*. Test for total alkaloids was carried out according to the gravimetric method as described by Senguttuvan, Paulsamy, and Karthika [13]. Briefly, to 5 g of sample, 200 mL of acetic acid (20%) was added, covered and left to stand for 4 h. This mixture was filtered, and the filtrate was reduced to a quarter (1/4) of the volume using a water bath. Concentrated ammonium hydroxide was added dropwise to the reduced filtrate until the precipitate was complete. The whole mixture was left to settle before filtration of the mixture was carried out, precipitate collected, and weighed. Total alkaloids were calculated according to equation (3):

(3) $$\begin{array}{c} \text{Total alkaloids }\left(\text{mg}/\text{g}\right)=\text{weight of residue}/\text{weight of sample taken}\times 100. \end{array}$$

The values were expressed as pg per g of extract (pg/g).

### 2.6. Antioxidant Assessment

#### 2.6.1. 2,2-Diphenyl-2-Picrylhydrazyl (DPPH) Radical Scavenging Assay

Scavenging activity of DPPH radical was assessed according to the method described by Sharma et al. [11]. Briefly, 500 *μ*L of both DPPH (0.1 M) and varying concentrations of standard/test solution (1-5 mg/mL) were pipetted in tubes and incubated at room temperature for 30 min in the dark. After incubation, the absorbance was recorded at 517 nm. Ascorbic acid and butylated hydroxytoluene (BHT) were used as standards, while dimethyl sulfoxide (DMSO) was used as control. The scavenging activity of the sample at each concentration was expressed as the percentage of DPPH scavenged and calculated using

(4) $$\begin{array}{c} \%\text{ DPPH Scavenged}=100\times \left(\frac{A_{c}-A_{s}}{A_{c}}\right), \end{array}$$

where *A*_*c*_ = absorbance of the control and *A*_*s*_ = absorbance of the test sample.

IC_50_ represents the concentration where 50% of radicals are scavenged by a test/standard sample.

#### 2.6.2. Hydrogen Peroxide (H_2_O_2_) Scavenging Assay

H_2_O_2_ radical scavenging activity was carried out according to the method described by Sharma et al. [11]. The solution of 40 mM H_2_O_2_ was prepared in phosphate buffer (50 mM, pH 7.4). Thereafter, 1.2 mL of different concentrations (1-5 mg/mL) of sample/standard and 0.6 mL of H_2_O_2_ solution were pipetted into a tube and incubated for 10 min at room temperature. The absorbance of the mixture at 230 nm was taken after incubation. Ascorbic acid and BHT were used as standards while phosphate buffer was used as control. The scavenging activity of the sample at each concentration was expressed in percentage of scavenged H_2_O_2_ and calculated using

(5) $$\begin{array}{c} \%\;{\text{H}}_{2}{\text{O}}_{2}\text{ Scavenged}=100\times \left(\frac{A_{c}-A_{s}}{A_{c}}\right), \end{array}$$

where *A*_*c*_ = absorbance of the control and *A*_*s*_ = absorbance of the test sample.

#### 2.6.3. Ferric Reducing Antioxidant Power (FRAP)

FRAP was carried out according to the method described by Sharma et al. [11]. To an aliquot of the sample (1 mL), 2.5 mL of 0.2 M phosphate buffer (pH 6.6) and 2.5 mL of 1% potassium ferricyanide were pipetted sequentially in a tube and the mixture was incubated at 50°C for 20 min. Afterwards, 2.5 mL of 10% trichloroacetic acid was added, mixture centrifuged for 10 min, and 2.5 mL of the supernatant obtained. Five hundred *μ*L of 0.1% FeCl_3_ and 2.5 mL of distilled H_2_O were added to the supernatant, mixed thoroughly, and absorbance of the mixture recorded at 700 nm. Values were expressed as mg of ascorbic acid equivalent per g of extract (mg AAE/g extract).

#### 2.6.4. Total Antioxidant Activity (TAC)

TAC was carried out according to the method described by Sharma et al. [11]. Briefly, 3 mL reagent solution (0.6 M sulphuric acid, 28 mM sodium phosphate, and 4 mM ammonium molybdate) was pipetted into tubes containing 300 *μ*L of extract. The reaction mixture was capped, boiled at 95°C for 90 min, and left to cool at room temperature. The absorbance was measured at 695 nm against blank (methanol 300 *μ*L). Ascorbic acid was taken as the standard, and values were expressed as mg of ascorbic acid equivalent per g of extract (mg AAE/g extract).

### 2.7. Anti-Inflammatory Assessment

#### 2.7.1. Albumin Denaturation Assay

Albumin denaturation protection using bovine serum albumin was assayed following the method described by Sharma et al. [11]. Briefly, 50 *μ*L of graded concentrations of samples/standard (1-5 mg/mL) was incubated with 5 mL of bovine serum albumin (BSA) solution (0.2 *w*/*v* prepared in Tris buffer (pH 6.8)). The samples were heated at 72°C for 5 min, cooled at room temperature for 15 min, and absorbance read at 660 nm. Ibuprofen was used as a standard, while methanol was used as control. The percentage inhibition of precipitation (denaturation of proteins) was determined following

(6) $$\begin{array}{c} \%\text{ denaturation inhibition}=100\times \left(\frac{A_{c}-A_{s}}{A_{c}}\right), \end{array}$$

where *A*_*c*_ = absorbance of the control and *A*_*s*_ = absorbance of the test sample.

#### 2.7.2. Hypotonic Solution-Induced Haemolysis Stabilising Assay

The method described by Sabiu and Ashafa [14] was adopted. In brief, 0.5 mL of erythrocyte (RBC) suspension was added to 4.5 mL of hypotonic solution (5 mM NaCl in sodium phosphate buffer (10 mM, pH 7.4)) containing 1 mL of extract/ibuprofen at varying concentration (1–5 mg/mL). For the control sample, 0.5 mL of RBCs was mixed with the hypotonic-buffered saline alone. The resulting mixture was incubated at 20°C for 10 min and subsequently centrifuged at 3000 rpm for 10 min. The absorbance was read at 540 nm. The percentage inhibition of either haemolysis or membrane stabilisation was calculated using

(7) $$\begin{array}{c} \%\text{ inhibition of haemolysis}=100\times \left(\frac{A_{c}-A_{s}}{A_{c}}\right), \end{array}$$

where *A*_*c*_ = absorbance of the control and *A*_*s*_ = absorbance of the test sample in hypotonic solution.

#### 2.7.3. Heat Solution-Induced Haemolysis Stabilising Assay

The method described by Sabiu and Ashafa [14] was adopted. An aliquot (4.5 mL) of isotonic buffer (0.25% *w*/*v* sodium chloride in 0.15 M sodium phosphate buffer (pH 7.4)) containing 0.5 mL of graded sample/standard concentration (1–5 mg/mL) was put into two duplicate sets of tubes. Thereafter, 0.5 mL of 2% (*v*/*v*) human erythrocyte suspension was added and gently mixed. One set of tubes was placed in a water bath at 54°C for 20 min, while the other set was placed on ice at 0–5°C at the same incubation time. The reaction mixtures were centrifuged for 5 min at 3000 rpm, and the absorbance of the supernatant was measured at 540 nm. Ibuprofen was used as a standard while distilled water was used as control. The percentage of haemolysis in test samples was calculated using

(8) $$\begin{array}{c} \%\text{ haemolysis}=100\times \left(\frac{A_{\text{th}}-A_{\text{tuh}}}{A_{c}-A_{\text{tuh}}}\right), \end{array}$$

where *A*_th_ = absorbance of test sample heated; *A*_tuh_ = absorbance of test sample unheated; *A*_*c*_ = absorbance of control sample heated.

### 2.8. Antidiabetic Assessment

#### 2.8.1. Alpha-Glucosidase (*α*-Glucosidase) Inhibitory Activity

*α*-Glucosidase inhibitory activity of the extracts was determined according to the method described by Iheagwam et al. [15]. Briefly, 250 *μ*L of each extract or acarbose at different concentrations (1-5 mg/mL) was incubated with 500 *μ*L of 1.0 U/mL *α*-glucosidase solution in 100 mM phosphate buffer (pH 6.8) at 37°C for 15 min. Thereafter, 250 *μ*L of *ρ*-Nitrophenyl-*α*-D-glucopyranoside (*ρ*NPG) solution (5 mM) in 100 mM phosphate buffer (pH 6.8) was added, and the mixture was further incubated at 37°C for 20 min. The absorbance of the released *p*-nitrophenol was measured at 405 nm, and the inhibitory activity was expressed as a percentage of control without inhibitors. The *α*-glucosidase inhibitory activity was calculated according to equation (9):

(9) $$\begin{array}{c} \%\text{ inhibition}=100\times \left(\frac{A_{c}-A_{s}}{A_{c}}\right), \end{array}$$

where *A*_*s*_ is the absorbance in the presence of sample and *A*_*c*_ is the absorbance of control.

#### 2.8.2. Alpha-Glucosidase (*α*-Glucosidase) Inhibitory Kinetics

For *α*-glucosidase inhibition kinetics, the method described by Iheagwam et al. [15] was adopted. Briefly, 250 *μ*L of extract (5 mg/mL) was preincubated with 500 *μ*L of *α*-glucosidase solution for 10 min at 25°C as inhibitor samples while 500 *μ*L of *α*-glucosidase was preincubated with 250 *μ*L of phosphate buffer (pH 6.9) in another set of tubes as noninhibitor samples. An aliquot (250 *μ*L) of *ρ*NPG at varying concentrations (0.15-5 mg/mL) was added to both sets of reaction mixtures to start the reaction. The mixture was then incubated for 10 min at 25°C, and 100 *μ*L of Na_2_CO_3_ was thereafter added to stop the reaction. The amount of reducing sugars released was determined spectrophotometrically using a *p*-nitrophenol standard curve. Reaction rates (V) were thereafter calculated, and double reciprocal plots of enzyme kinetics were constructed according to Lineweaver-Burk method to study the nature of inhibition. K_m_ and V_max_ values were also calculated from the Lineweaver-Burk plot.

#### 2.8.3. Alpha-Amylase (*α*-Amylase) Inhibitory Activity

A volume of 250 *μ*L of each fraction or acarbose at different concentrations (1–5 mg/mL) was incubated with 500 *μ*L of porcine pancreatic amylase (2 U/mL) in 100 mM phosphate buffer (pH 6.8) at 37°C for 20 min. An aliquot (250 *μ*L) of 1% starch solution (dissolved in 100 mM phosphate buffer (pH 6.8)) was then added to the reaction mixture and incubated at 37°C for 1 h. One mL of DNS colour reagent was then added and boiled for 10 min. The absorbance of the resulting mixture was measured at 540 nm, and the inhibitory activity was expressed as a percentage of control without inhibitors [15]. The *α*-amylase inhibitory activity was calculated using equation (10):

(10) $$\begin{array}{c} \%\text{ inhibition}=100\times \left(\frac{A_{c}-A_{s}}{A_{c}}\right), \end{array}$$

where *A*_*s*_ is the absorbance in the presence of sample and *A*_*c*_ is the absorbance of control.

#### 2.8.4. Alpha-Amylase (*α*-Amylase) Inhibitory Kinetics

For the kinetic experiments, 250 *μ*L of extract (5 mg/mL) was taken and incubated with 500 *μ*L of *α*-amylase (2 U/mL) in 100 mM phosphate buffer (pH 6.8), prior to the addition of 250 *μ*L of varied concentration of starch (0.3-10 mg/mL). The reaction was allowed to proceed as highlighted above. The Lineweaver-Burk double reciprocal plot (1/V versus 1/[S]) was constructed, and the kinetics of *α*-amylase inhibition by the extract was determined [15].

### 2.9. Statistical Analysis

All data were subjected to one-way analysis of variance (ANOVA) followed by Duncan posttest. A 5% probability level was considered significant as values were represented as mean ± standard deviation (SD) of three (3) replicates. Linear regression and Pearson correlation analysis were also employed to identify the association between antioxidant activity and antidiabetic properties. These analyses were run using IBM SPSS Statistics 23 (IBM Corp., New York, USA).

## 3. Result

### 3.1. Phytochemical Analysis

The preliminary phytochemical analysis revealed that both *N. latifolia* leaf extracts contained phenols, terpenoids, cardiac glycosides, quinones, alkaloids, flavonoids, saponins, and tannins, while coumarins and glycosides were absent. However, anthocyanins and betacyanins were detected only in NLE, while triterpenoids were present in NLA only (Table 1).

NLE extract demonstrated significantly (*p* < 0.05) higher tannin, alkaloid, *β*-carotene, and lycopene contents than NLA by 8.52, 184.21, 126.67, and 140.68%, respectively (Table 2). Nonetheless, there was no significant (*p* > 0.05) difference in total flavonoid and phenolic content.

### 3.2. Antioxidant Assessment

**Figure 1:**
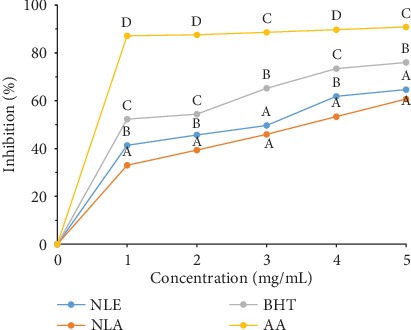
DPPH radical scavenging ability of *N. latifolia* leaf extracts and standards. Data are represented as mean ± SD (*n* = 3). Points on a concentration with the same superscript alphabet indicate no significant difference while different superscript alphabet indicates significant difference (*p* < 0.05).

The data of *N. latifolia* extracts and standards radical scavenging ability on DPPH radical were concentration-dependent as shown in Figure 1. The ethanolic extract exhibited a significantly (*p* < 0.05) higher percentage inhibition compared to the aqueous extract at all concentrations except for 5 mg/mL. This was evident with an IC_50_ (2.58 ± 0.08 mg/mL) significantly (*p* < 0.05) lower to that of the aqueous extract (3.51 ± 0.05 mg/mL). Furthermore, the IC_50_ of butylated hydroxytoluene (BHT) (0.92 ± 0.03 mg/mL) and ascorbic acid (AA) (0.08 ± 0.01 mg/mL) were way (*p* < 0.05) lower than the extracts (Table 3).

**Figure 2:**
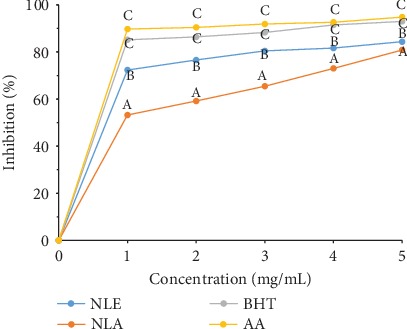
H_2_O_2_ radical scavenging ability of *N. latifolia* leaf extracts and standards. Points on a concentration with the same superscript alphabet indicate no significant difference while different superscript alphabet indicates significant difference (*p* < 0.05).

A similar observation was also made in Figure 2, where the scavenging of hydrogen peroxide radical was dependent on the concentration of the extracts with NLE having a significantly (*p* < 0.05) higher inhibitory activity than NLA. This was further buttressed by their IC_50_ values as both NLE (0.39 ± 0.08 mg/mL) and NLA (0.96 ± 0.09 mg/mL) were significantly lower than BHT (0.02 ± 0.00 mg/mL) and AA (0.01 ± 0.00 mg/mL) (Table 3).

It was observed that TAC and FRAP exhibited by NLE were significantly (*p* < 0.05) higher than NLA in all concentrations (Table 4).

### 3.3. Anti-Inflammatory Assessment

*N. latifolia* extracts palliated the denaturation of albumin in a dose-dependent manner. However, the level of inhibition elicited by the extracts was significantly (*p* < 0.05) lower than that observed for ibuprofen (Table 5).

The extracts exhibited a dose-dependent inhibition of hypotonic solution-induced haemolysis of erythrocyte membrane. Both NLE and NLA had significantly (*p* < 0.05) higher hypotonic solution-induced haemolysis than ibuprofen at all graded concentrations (Table 6). Results from Table 7 showed a scaling increase in both extract and standard dosage reduced the level of heat-induced haemolysis. The inhibition of haemolysis potentiated by *N. latifolia* extracts were significantly (*p* < 0.05) lower than ibuprofen at all doses.

### 3.4. Antidiabetic Assessment

**Figure 3:**
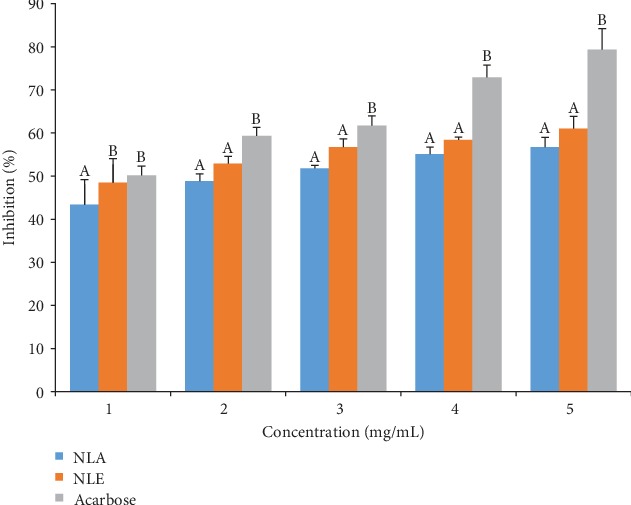
Inhibitory activity of *N. latifolia* leaf extracts on *α*-amylase activity. Bars are expressed as means ± SD of triplicate determinations. Values with different superscripts on each concentration are significantly different (*p* < 0.05).

**Figure 4:**
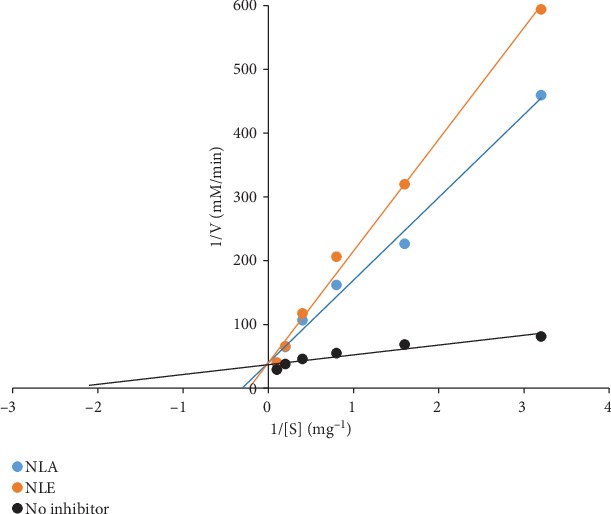
Mechanism of inhibition of *α*-amylase by *N. latifolia* leaf extracts.

**Figure 5:**
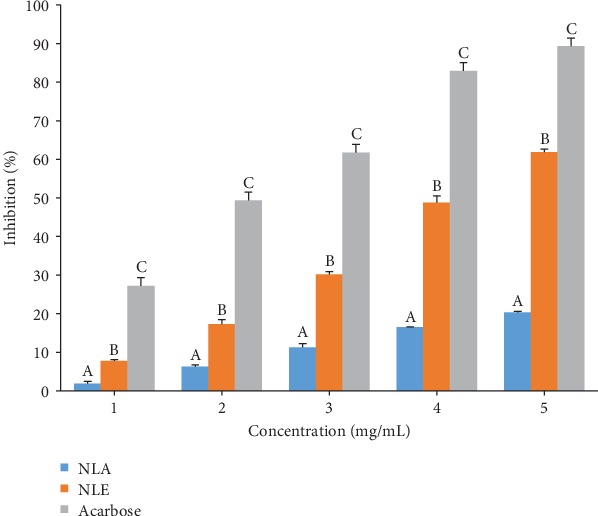
Inhibitory activity of *N. latifolia* leaf extracts on *α*-glucosidase activity. Bars are expressed as mean ± SD of triplicate determinations. Values with different superscripts on each concentration are significantly different (*p* < 0.05).

**Figure 6:**
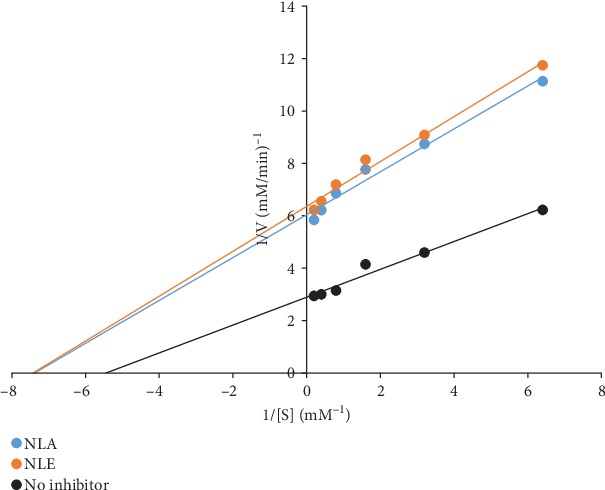
Mechanism of inhibition of *α*-glucosidase by *N. latifolia* leaf extracts.

*α*-Amylase inhibitory activity by *N. latifolia* extracts as depicted in Figure 3 shows a concentration-dependent inhibitory effect was observed, with NLE eliciting inhibitory effect that competed favourably with acarbose at the lowest concentration. A contrary observation was recorded for higher concentrations where the NLE and NLA had a significantly (*p* < 0.05) lower percentage inhibitions. *N. latifolia* aqueous and ethanol extracts displayed higher IC_50_ (2.64 ± 0.48 and 1.19 ± 0.11, respectively) compared to acarbose (0.85 ± 0.18) (Table 8). The extracts also displayed a competitive inhibition on *α*-amylase activity (Figure 4), with a common *V*_max_ of 0.025 mM/min and *K*_m_ of 3.33 and 5 mg for NLA and NLE, respectively, from the Lineweaver-Burk plot (Table 8). For *α*-glucosidase activity, a significant (*p* < 0.05) difference on inhibition of enzyme activity by the extracts and standard was elicited across concentrations (Figure 5). NLE (4.20 ± 0.18 mg/mL) and NLA (11.21 ± 0.35 mg/mL) exhibited higher IC_50_ than acarbose (2.23 ± 0.21 mg/mL) further buttressing the observation (Table 8). The kinetic study using the Lineweaver-Burk plot revealed the extracts inhibited *α*-glucosidase activity uncompetitively (Figure 6). The K_m_ of 0.14 mM and V_max_ of 0.17 and 0.16 mM/min for NLA and NLE, respectively, were extrapolated (Table 9).

From the regression analysis, a significant (*p* > 0.05, *p* > 0.01) positive correlation was observed between the antioxidant activity elicited by *N. latifolia* leaf extracts and their carbohydrate-metabolising enzyme inhibitory activity showing an association between these two activities (Tables 9 and 10).

Values under Pearson correlation and linear regression represent correlation coefficient and *p* value, respectively. ^∗∗^Correlation is significant at 0.01 level (2-tailed). ^∗^Correlation is significant at 0.05 level (2-tailed).

## 4. Discussion

Medicinal plants are of value to mankind due to their rich contents in a wide range of minerals, primary and secondary metabolites, which have numerous reported therapeutic properties [16]. Phyto biocompounds such as phenolic compounds, flavonoids, alkaloids, and terpenes are known to possess anticancer, antioxidant, antiviral, and other protective abilities. They also scavenge free radicals and modify cellular events [17]. These phytochemicals were widely detected in both extracts due to their solubility and polarity, supporting the use of *N. latifolia* leaves by the locals in treating diverse ailments and diseases [18, 19]. The findings were corroborated by a review reported by Balogun, Besong, Obu, Obu, and Djobissie [20], as most of these plant metabolites were also detected in the leaves. Interestingly, a contrary finding was made in this study as glycoside was absent. The higher amount of the studied secondary metabolites in NLE could be attributed to the ability of ethanol to easily permeate the membrane and extract intracellular ingredients from plant materials [21]. The increased TTC in the ethanol extracts may be due to lower dissolving attributes of nonphenol compounds such as carbohydrate and terpenes in ethanol than in water. A concomitant formation of complex phenolic compounds soluble in ethanol may occur [21]. Higher *β*-carotene and lycopene content in NLE is as a result of their classification and structure making them more soluble in ethanol than water [22, 23]. Previous studies have reported a higher phytochemical content in their ethanolic extracts than their aqueous thus corroborating the findings in this study [15, 24]. Antioxidants scavenge free radicals which are products of metabolism. Phenolic compounds such as phenols, flavonoids, and tannins have been proven to efficiently act as antioxidants in medicinal plants [25]. The mechanism of antioxidant action is by donating hydrogen to reactive oxygen and nitrogen species thereby quenching and scavenging them. They also chelate metals, and when consumed, reduce the incidence of disease and promote health [26]. The result was suggestive of the antioxidant potential *N. latifolia* leaves possess as reported by previous finding [27]. Though moderate to good antioxidant activity was observed for all extracts, the higher antioxidant capacity and scavenging activity of NLE may be as a result of higher tannin contents. This agrees with the previous findings from Alimi and Ashafa [17] and Unuofin, Otunola, and Afolayan [28], where ethanolic extracts scavenged more radicals than aqueous extracts. The reported DPPH radical scavenging activity and FRAP potential in this study were higher than previous studies on *N. latifolia* parts [29, 30]. The IC_50_ of ascorbic acid on DPPH was smaller in our study compared to other previous studies [31, 32]. Assessment of protein denaturation and erythrocyte membrane stabilization are some methods anti-inflammatory potentials of compounds can be tested [11]. When erythrocytes are subjected to external cellular factors such as heat and hypotonic medium, biomembrane lysis occurs with autooxidation of the released free haemoglobin and haemolysis as a concomitant result [33]. Disturbance of biomembrane integrity, as well as its break down, is attributed to increased cellular damage and free radicals such as malondialdehyde. This instance is a common phenomenon in diseases such as diabetes mellitus [34]. Medicinal plants boost biomembrane stability by reducing lysosomal enzyme activity, maintaining cell membrane fluidity and ion gradients [35, 36]. The observed membrane-stabilizing potential of *N. latifolia* extracts may be as a result of the ability of the phytochemicals present in the extracts to bind with the erythrocyte membrane preventing disruption of membrane integrity by alteration of surface charges. The higher anti-inflammatory property elicited by NLE may be attributed to the presence of more tannin, lycopene, and *β*-carotene content. The anti-inflammatory activity of this class of phytochemical has been well reported such as the erythrocyte membrane stabilising effect, cation-binding activity of tannins and wound healing properties of lycopene and *β*-carotene [36–38]. Boucherle et al. [39] reported the presence of tannins in the leaf extract of *N. latifolia* buttressing findings in this report. Different researches have attributed the anti-inflammatory activity of their plants to phytochemical action on membrane via membrane binding, membrane fluidity maintenance, and ion gradient maintenance corroborating our results [14, 33, 35]. The inhibition of major enzymes that metabolise carbohydrate activity such as *α*-glucosidase and *α*-amylase has been used as a therapeutic control. This delays carbohydrate digestion time, reduces glucose absorption rate, ultimately controlling postprandial hyperglycaemia [40, 41]. Phyto-derived inhibitors of these carbohydrate-metabolising enzymes with excellent antioxidative potentials are a great complementary alternative in potentiating adverse effects such as flatulence and diarrhoea, which results from the fermentation of undigested carbohydrate in the colon usually witnessed with synthetic inhibitors such as acarbose and voglibose [15, 42]. This study revealed the promising inhibitory *α*-amylase and *α*-glucosidase potential of *N. latifolia* leaf extracts. A previous report on *N. latifolia* whole fruit extract fractions inhibitory action on *α*-amylase had similar IC_50_ values, while *α*-glucosidase IC_50_ values were way lower than what this study reported [43]. Interestingly, the IC_50_ of acarbose on *α*-amylase and *α*-glucosidase in this study was, respectively, lower and higher than the reported values by Oyedeji-Amusa and Ashafa [43] and Akanji, Olukolu, and Kazeem [44]. The mild inhibition of *N. latifolia* leaf extracts compared to acarbose is suggestive of a lower probability to cause various side effect associated with the inhibition of these enzymes [45]. From kinetic studies, the extracts exerted two modes of inhibitions for *α*-glucosidase and *α*-amylase, namely, the uncompetitive and competitive mechanisms, respectively. Competitive inhibitors bind to the enzymes active site, displacing the substrate to form enzyme-inhibitor complex (EI), while uncompetitive inhibitors bind to enzyme-substrate (ES) complex forming an enzyme-substrate-inhibitor (ESI) complex. The formation of the (ES) complex may cause a rearrangement of the enzyme conformation making it favourable for the bioactives in *N. latifolia* leaf extracts to bind to the allosteric site and inhibit *α*-glucosidase activity uncompetitively [46]. In contrast, they may be in direct competition with the substrate for the inhibition of *α*-amylase. This is the first time to the best of our knowledge, this has been reported on the mechanism of inhibition of *N. latifolia* leave extracts on the activity of key carbohydrate-metabolising enzymes linked to diabetes mellitus. The antioxidant activity of *N. latifolia* leave extracts displayed an association with carbohydrate-metabolising enzymes inhibitory activity by having a positive correlation. Previous studies have shown a positive correlation between the antioxidant properties of their extract to the amount of secondary plant metabolites present [9, 47] and the carbohydrate-metabolising enzymes inhibitory activity [48]. On the contrary, Ademiluyi, Oboh, Aragbaiye, Oyeleye, and Ogunsuyi [49], in their reports, showed no correlation between the antioxidant properties and carbohydrate-metabolising enzymes inhibitory activity of their plant. They suggested the extracts inhibitory activity could be attributed to phenolic and nonphenolic phytochemicals interactions with the enzyme. Suppression of oxidative stress is a potential mechanism of natural products in treating diabetes [50]. A compound's reducing capacity is linked to its antioxidant activity [51]; thus, the observed inhibitory property may be as a result of its antioxidant activities. Sales, Souza, Simeoni, Magalhães, and Silveira [52] have reported flavonoids and other phenolic compounds demonstrate high *α*-amylase inhibitory activity due to the presence of hydroxyl groups.

## 5. Conclusions

This research data provides an insight into the antioxidant, anti-inflammatory, and antidiabetic potential of *N. latifolia* leaf extracts as well as the relationship between these activities. The leaf is a rich source of natural antioxidant, anti-inflammatory, and antidiabetic compounds. It further suggests its relevance in the management of postprandial hyperglycaemia by suppressing the hydrolysis of dietary carbohydrates. The bioactive compounds responsible for this antioxidant, anti-inflammatory, and antidiabetic activities were not identified and thus, could be identified, isolated, and assessed. Also, *in vivo* confirmation of *N. latifolia* leaf antidiabetic potential and mechanisms should be further evaluated.

## Figures and Tables

**Table 1:** Qualitative phytochemical constituents of *N. latifolia* leaf extracts.

Phytochemical	NLE	NLA
Tannins	+ve	+ve
Saponins	+ve	+ve
Flavonoids	+ve	+ve
Alkaloids	+ve	+ve
Anthocyanin	+ve	-ve
Betacyanins	+ve	-ve
Quinones	+ve	+ve
Glycosides	-ve	-ve
Cardiac glycosides	+ve	+ve
Terpenoids	+ve	+ve
Triterpenoids	-ve	+ve
Phenols	+ve	+ve
Coumarins	-ve	-ve

+ve: detected; −ve: not detected.

**Table 2:** Quantitative phytochemical constituents of *N. latifolia* leaf extracts.

Phytochemical	NLE	NLA
TFC (mg RE/g)	11.02 ± 0.25^a^	8.57 ± 0.13^a^
TPC (mg GAE/g)	55.68 ± 0.45^a^	54.07 ± 2.64^a^
TTC (mg TAE/g)	183.96 ± 7.68^b^	169.51 ± 6.20^a^
*β*-Carotene (mg CE/g)	0.108 ± 0.05^b^	0.038 ± 0.02^a^
Lycopene (mg CE/g)	0.034 ± 0.03^b^	0.015 ± 0.04^a^
Total alkaloid (mg/g)	323.45 ± 5.80^b^	134.39 ± 3.52^a^

“TFC,” “TPC,” and “TTC” mean total flavonoid content, total phenolic content, and total tannin content, respectively. “mg RE/g”: mg rutin equivalents per gram extract; “mg GAE/g”: mg gallic acid equivalents per gram extract; “mg TAE/g”: mg tannic acid equivalents per gram extract; “mg CE/g”: mg carotenoid equivalents per gram extract; “mg/g”: mg per gram extract. Data are represented as mean ± SD (*n* = 3). Values across the same row with the same superscript alphabet indicate no significant difference while different superscript alphabet indicates significant difference (*p* < 0.05).

**Table 3:** IC_50_ values for DPPH and H_2_O_2_ radical scavenging ability of *N. latifolia* leaf extracts and standards.

Concentration (mg/mL)	IC_50_			
	NLE	NLA	BHT	AA
DPPH	2.58 ± 0.08^b^	3.51 ± 0.05^a^	0.92 ± 0.03^c^	0.08 ± 0.01^d^
H_2_O_2_	0.39 ± 0.08^b^	0.96 ± 0.09^c^	0.02 ± 0.00^a^	0.01 ± 0.00^a^

“BHT” and “AA” mean butylated hydroxytoluene and ascorbic acid, respectively. Data are mean ± SD (*n* = 3). Values across the same row with the same superscript alphabet indicate no significant difference while different superscript alphabet indicates significant difference (*p* < 0.05).

**Table 4:** Total antioxidant capacity and ferric reducing antioxidant power of *N. latifolia* leaf extracts.

Concentration (mg/mL)	TAC (*μ*g AAE/g)		FRAP (*μ*g AAE/g)	
	NLE	NLA	NLE	NLA
1	2.95 ± 0.22^a^	1.33 ± 0.16^b^	89.10 ± 5.55^c^	80.96 ± 2.68^d^
2	12.97 ± 0.59^a^	6.85 ± 0.23^b^	325.70 ± 12.38^c^	219.60 ± 15.22^d^
3	28.62 ± 0.55^a^	17.14 ± 1.36^b^	636.63 ± 34.90^c^	434.09 ± 13.72^d^
4	51.37 ± 2.03^a^	30.11 ± 3.72^b^	964.03 ± 77.67^c^	764.40 ± 43.35^d^
5	73.81 ± 2.27^a^	55.63 ± 5.03^b^	1314.45 ± 71.64^c^	1044.44 ± 31.15^d^

“TAC,” “FRAP,” and “*μ*g AAE/g” mean total antioxidant capacity, ferric reducing antioxidant power, and *μ*g ascorbic acid equivalents per gram extract, respectively. Data are mean ± SD (*n* = 3). Values with different superscripts across the same row are significantly different (*p* < 0.05).

**Table 5:** Inhibitory effect of *N. latifolia* leaf extracts on albumin denaturation.

Concentration (mg/mL)	Inhibition (%)		
	NLE	NLA	Ibuprofen
1	16.45 ± 1.71^a^	13.56 ± 1.93^a^	37.82 ± 2.94^b^
2	26.68 ± 2.48^a^	21.08 ± 1.78^a^	46.63 ± 1.23^b^
3	34.82 ± 0.94^a^	39.12 ± 1.13^a^	64.08 ± 3.19^b^
4	49.82 ± 1.54^a^	58.03 ± 2.69^a^	85.03 ± 2.67^b^
5	70.54 ± 2.45^a^	68.05 ± 1.03^a^	92.04 ± 1.23^b^

Data are mean ± SD (*n* = 3). Values with different superscripts across the same row are significantly different (*p* < 0.05).

**Table 6:** Inhibitory effect of *N. latifolia* leaf extracts on hypotonic solution-induced haemolysis of erythrocyte membrane.

Concentration (mg/mL)	Haemolysis (%)		
	NLE	NLA	Ibuprofen
1	76.37 ± 6.64^a^	89.17 ± 7.02^b^	7.23 ± 1.84^c^
2	63.45 ± 1.27^a^	72.92 ± 7.63^b^	6.44 ± 1.05^c^
3	35.59 ± 3.26^a^	50.36 ± 1.06^b^	3.91 ± 0.32^c^
4	26.51 ± 2.19^a^	30.83 ± 3.73^b^	3.03 ± 0.68^c^
5	16.07 ± 1.60^a^	20.59 ± 4.60^b^	2.21 ± 0.83^c^

Data are mean ± SD (*n* = 3). Values with different superscripts across the same row are significantly different (*p* < 0.05).

**Table 7:** Inhibitory effect of *N. latifolia* leaf extracts on heat-induced haemolysis of erythrocyte membrane.

Concentration (mg/mL)	Haemolysis (%)		
	NLE	NLA	Ibuprofen
1	61.37 ± 6.64^a^	81.33 ± 6.64^b^	37.41 ± 0.41^c^
2	49.21 ± 1.27^a^	78.99 ± 1.27^b^	32.78 ± 3.13^c^
3	40.91 ± 3.26^a^	74.72 ± 3.26^b^	13.55 ± 2.99^c^
4	25.16 ± 2.19^a^	42.90 ± 2.19^b^	7.93 ± 1.07^c^
5	14.08 ± 1.76^a^	24.07 ± 1.06^b^	5.29 ± 0.41^c^

Data are mean ± SD (*n* = 3). Values with different superscripts across the same row are significantly different (*p* < 0.05).

**Table 8:** IC_50_, *V*_max_, and *K*_m_ values for the inhibition of carbohydrate-metabolising enzymes by *N. latifolia* leaf extracts.

	NLE	NLA	Acarbose	No inhibitor
*α*-Amylase				
IC_50_	1.19 ± 0.11^b^	2.64 ± 0.48^a^	0.85 ± 0.18^c^	—
*R*^2^	0.9696	0.9626	0.9745	—
*V*_max_ (mM/min)	0.03	0.03	—	0.03
K_m_ (mg)	5.00^c^	3.33^b^	—	0.43^a^
*α*-Glucosidase				
IC_50_	4.20 ± 0.18^b^	11.21 ± 0.35^c^	2.23 ± 0.21^a^	—
*R*^2^	0.9888	0.9979	0.9746	—
*V*_max_ (mM/min)	0.16^a^	0.17^a^	—	0.35^b^
*K*_m_ (mM)	0.14	0.14	—	0.19

Values with different superscripts across the same row are significantly different (*p* < 0.05).

**Table 9:** Association between antioxidant activity and carbohydrate-metabolising enzyme inhibitory activity of *N. latifolia* ethanol leaf extract.

Dependent variable	Pearson correlation				Linear regression			
	DPPH	H_2_O_2_	TAC	FRAP	DPPH	H_2_O_2_	TAC	FRAP
*α*-Glucosidase	0.981^∗∗^	0.979^∗∗^	0.986^∗∗^	0.996^∗∗^	0.003	0.004	0.002	0.000
*α*-Amylase	0.949^∗^	0.990^∗∗^	0.942^∗^	0.996^∗∗^	0.014	0.001	0.016	0.008

**Table 10:** Association between antioxidant activity and carbohydrate-metabolising enzyme inhibitory activity of *N. latifolia* aqueous leaf extract.

Dependent variable	Pearson correlation				Linear regression			
	DPPH	H_2_O_2_	TAC	FRAP	DPPH	H_2_O_2_	TAC	FRAP
*α*-Glucosidase	0.997^∗∗^	0.998^∗∗^	0.972^∗∗^	0.998^∗∗^	0.000	0.000	0.006	0.000
*α*-Amylase	0.978^∗^	0.972^∗∗^	0.911^∗^	0.949^∗^	0.004	0.005	0.031	0.014

Values under Pearson correlation and linear regression represent correlation coefficient and *p* value, respectively. ^∗∗^Correlation is significant at 0.01 level (2-tailed). ^∗^Correlation is significant at 0.05 level (2-tailed).

## Data Availability

The data used to support the findings of this study are included in the article.
